# Notch Signaling in Descending Thoracic Aortic Aneurysm and Dissection

**DOI:** 10.1371/journal.pone.0052833

**Published:** 2012-12-26

**Authors:** Sili Zou, Pingping Ren, Mary Nguyen, Joseph S. Coselli, Ying H. Shen, Scott A. LeMaire

**Affiliations:** 1 Division of Cardiothoracic Surgery, Michael E. DeBakey Department of Surgery, Baylor College of Medicine, Houston, Texas, United States of America; 2 Department of Cardiovascular Surgery, Texas Heart Institute at St. Luke's Episcopal Hospital, Houston, Texas, United States of America; 3 Department of Vascular Surgery, Changhai Hospital, Second Military Medical University, Shanghai, China; University of Illinois at Chicago, United States of America

## Abstract

**Background:**

Descending thoracic aortic aneurysm and dissection (DTAAD) is characterized by progressive medial degeneration, which may result from excessive tissue destruction and insufficient repair. Resistance to tissue destruction and aortic self-repair are critical in preventing medial degeneration. The signaling pathways that control these processes in DTAAD are poorly understood. Because Notch signaling is a critical pathway for cell survival, proliferation, and tissue repair, we examined its activation in DTAAD.

**Methods:**

We studied descending thoracic aortic tissue from patients with sporadic thoracic aortic aneurysm (TAA; n = 14) or chronic thoracic aortic dissection (TAD; n = 16) and from age-matched organ donors (n = 12). Using western blot, real-time RT-PCR, and immunofluorescence staining, we examined aortic tissue samples for the Notch ligands Delta-like 1, Delta-like 4 (DLL1/4), and Jagged1; the Notch receptor 1 (Notch1); the Notch1 intracellular domain (NICD); and Hes1, a downstream target of Notch signaling.

**Results:**

Western blots and RT-PCR showed higher levels of the Notch1 protein and mRNA and the NICD and Hes1 proteins in both TAA and TAD tissues than in control tissue. However, immunofluorescence staining showed a complex pattern of Notch signaling in the diseased tissue. The ligand DLL1/4 and Notch1 were significantly decreased and NICD and Hes1 were rarely detected in medial vascular smooth muscle cells (VSMCs) in both TAA and TAD tissues, indicating downregulation of Notch signaling in aortic VSMCs. Interestingly Jagged1, NICD, and Hes1 were highly present in CD34+ stem cells and Stro-1+ stem cells in aortas from TAA and TAD patients. NICD and Hes1 were also detected in most fibroblasts and macrophages that accumulated in the aortic wall of DTAAD patients.

**Conclusions:**

Notch signaling exhibits a complex pattern in DTAAD. The Notch pathway is impaired in medial VSMCs but activated in stem cells, fibroblasts, and macrophages.

## Introduction

Aortic aneurysm and dissection (AAD) account for almost 11,000 deaths in the United States each year [1]. Despite improvements in diagnostic and therapeutic techniques for AAD, the mortality rate remains high. Characterized by aortic medial degeneration, AAD presents as the progressive loss of smooth muscle cells (SMCs) [2] and the destruction of extracellular matrix [3]. Medial degeneration of the aorta leads to progressive aortic dilatation, and ultimately, to dissection or aneurysm rupture [4]. The overproduction of destructive factors plays a significant role in aortic degeneration and AAD development. In addition, impaired aortic protection (resistance to tissue destruction) and insufficient aortic repair may contribute to the process. However, the signaling mechanisms that control aortic protection and repair in AAD are poorly understood.

Notch signaling plays an important role in regulating tissue development and homeostasis [5], [6], [7] by controlling cell fate and specifying tissue patterning [8], [9], [10]. The Notch signaling pathway is activated by the binding of Delta-like or Jagged ligands to Notch receptors, and this binding triggers the ADAM protease-mediated cleavage of the Notch receptor extracellular domain. The subsequent γ-secretase–mediated cleavage of the Notch receptor releases the Notch1 intracellular domain (NICD), which translocates into the nucleus and regulates the expression of downstream genes [11], such as Hes1 [12].

Specifically, Notch signaling is important in controlling vascular smooth muscle cell (VSMC) differentiation [13], [14], and the pathway is critical to vascular development, repair, and remodeling [15], [16], [17], [18]. Recently, Notch signaling has been shown to be downregulated in human abdominal aortic aneurysm (AAA) tissue [19] and in the ascending aorta of patients with bicuspid aortic valve (BAV) [20]. Furthermore, genetic variation in the *NOTCH1* gene appears to confer susceptibility to ascending aortic aneurysm formation in patients with BAV [21]. However, Notch signaling has not been examined in sporadic descending thoracic aortic aneurysm and dissection (DTAAD).

Because of its important role in vascular repair and remodeling, we hypothesize that Notch signaling may be altered in DTAAD. In this study, we examined the activation of the Notch signaling pathway in aortic tissue from patients with DTAAD.

## Materials and Methods

### Patient enrollment and tissue collection

This study protocol was approved by the institutional review board at Baylor College of Medicine. Informed, written consent was obtained from all patients. We enrolled patients who underwent elective surgical repair of either a descending thoracic aortic aneurysm without dissection (TAA) or a chronic descending thoracic aortic dissection (TAD). We excluded patients who had acute symptoms (<14 days); BAV; heritable connective tissue disease (eg, Marfan syndrome); DTAAD related to trauma, aortitis, or infection; and first-degree relatives who had TAA or TAD. We obtained samples of aortic tissue from 30 DTAAD patients undergoing surgical repair: TAA patients (n = 14) and TAD patients (n = 16). In the latter group, the mean interval between the onset of dissection and operation was 5.1±4.5 years. During TAA and TAD repair, samples of the posterolateral aortic wall were excised from the site of maximal aortic dilatation; in cases of aortic dissection, these samples comprised the outer wall of the false lumen. After excision, samples were rinsed with cold saline, and any attached thrombus was removed. Control samples (International Institute for the Advancement of Medicine, Jessup, PA) of descending thoracic aortic tissue were obtained from 12 age-matched organ donors without aortic aneurysm, dissection, coarctation, or previous aortic repair. For protein extraction, tissues were snap-frozen in liquid nitrogen and stored at −80°C. For tissue staining, samples were fixed in formalin and embedded in paraffin. Table 1 shows the characteristics of the enrolled subjects. Compared with control subjects, DTAAD patients were more likely to have a history of smoking and hypertension.

**Table 1:** Patient characteristics.^a^

Characteristics	Control	TAA	TAD	*p*
	(n = 12)	(n = 14)	(n = 16)	Values
Age (y)	59.1±8.2	64.8±5.5	63.8±5.6	0.07
Men	4 (33%)	6 (43%)	11 (69%)	0.1
History of smoking	6 (50%)	14 (100%)	12 (75%)	0.01
Hypertension	6 (50%)	13 (93%)	16 (100%)	0.001
Diabetes mellitus	4 (33%)	2 (14%)	1 (6%)	0.2
Taking anti-lipid medication	2 (17%)	5 (36%)	5 (31%)	0.5
Aortic diameter at sample site (cm)	NA	6.4±0.9	6.3±1.3	0.9

a Age and aortic diameter were compared by using one-way analysis of variance. All other variables were compared by using Pearson's chi-squared test.

NA = not applicable; TAA = thoracic aortic aneurysm; TAD = thoracic aortic dissection.

### Western blot

Frozen aortic tissues (approximately 100 mg) were ground and homogenized. Protein content was extracted with RIPA buffer (Cell Signaling Technology, Danvers, MA), and protein concentration was determined by using the Bradford assay (Bio-Rad Laboratories, Hercules, CA). A total of 20 µg protein was electrophoretically separated in 4–20% Mini-PROTEAN TGX Gels (Bio-Rad Laboratories) and transferred onto polyvinylidene difluoride membranes (Bio-Rad Laboratories). Anti-Notch1 rabbit monoclonal antibody (1∶1000, Cell Signaling Technology), anti-cleaved Notch1 rabbit monoclonal antibody (1∶1000, Cell Signaling Technology), anti-Hes1 polyclonal antibody (1∶1000, EMD Millipore, Billerica, MA), and a horseradish peroxidase-labeled anti-rabbit secondary antibody (1∶5000, Santa Cruz Biotechnology, Santa Cruz, CA) were used to detect Notch1, NICD, and Hes1 proteins in the extract. An anti-β-actin mouse monoclonal antibody (1∶5000; Cell Signaling Technology) was used to confirm equal loading. Detailed information on the primary antibodies is provided in Table 2. The western blot bands were scanned and analyzed by using ImageJ software (National Institutes of Health).

**Table 2:** Primary antibodies used in the study.

Antibody	Source	Host	Specificity	Application
Anti-cleaved Notch1 (D3B8)	4147 Cell Signaling	Rabbit	Cleaved Notch1 intracellular domain (NICD) (∼110 kDa)	WB
Anti-Notch1 (D6F11)	4380 Cell Signaling	Rabbit	Full-length (∼300 kDa) and the transmembrane/intracellular region NTM (∼120 kDa)	WB
Anti-Stro-1	MAB1038 R&D	Mouse	Human Stro-1	IF
Anti-CD34 (ICO115)	3569 Cell Signaling	Mouse	Total CD34 protein	IF
Anti-SM22-α	ab14106 abcam	Rabbit	SM22-α	IF
Anti-SM22-α	ab10135 abcam	Goat	SM22-α	IF
Anti-ER-TR7	sc-73355 Santa Cruz	Rat	Fibroblasts	IF
Anti-CD68	ab955 Abcam	Mouse	Macrophage antigen CD68	IF
Anti-Notch1 (C-20)	sc-6014 Santa Cruz	Goat	C-terminus of Notch 1	IF
Anti-activated Notch1	ab8925 Abcam	Rabbit	Cleaved Notch1 intracellular domain (NICD)	IF
Anti-Jagged1 (C-20)	sc-6011 Santa Cruz	Goat	Jagged1	IF
Anti-Delta (C-20)	sc-8155 Santa Cruz	Goat	Delta-like 1 and Dleta-like 4 (DLL1/4)	IF
Anti-Hes1	AB5702 Millipore	Rabbit	Hes-1	IHC, IF

WB = western blot; IF = immunofluorescence staining; IHC = immunohistochemistry.

### Quantitative Real-time PCR

Total RNA was isolated with the Trizol (Invitrogen) method, and cDNA was synthesized with iScrip cDNA Synthesis Kit (Bio-rad Laboratories) from 1 µg of total RNA. Real-time PCR was performed with the cDNA samples and SYBR Green Supermix (Bio-rad Laboratories) by using a Bio-Rad iCycler & iQ Real-Time PCR Systems (Bio-rad Laboratories), and the formation of PCR products was monitored by using the SYBR green method. All samples were amplified in triplicate. The relative changes in the amount of transcripts in each sample were determined by normalizing with the 18S ribosomal RNA levels. The sequences of the primers for Notch1 used in real-time PCR were as follows: forward primer 5′-GCAGTTGTGCTCCTGAAGAA-3′; reverse primer 5′-CGGGCGGCCAGAAAC-3′.

### Double immunofluorescence staining

For double immunofluorescence staining, paraffin-embedded tissues were cut into 4 µm sections, deparaffinized, rehydrated, and then subjected to antigen retrieval. Tissue sections were incubated with primary antibodies overnight at 4°C, followed by incubation with secondary antibodies for 1 hour at room temperature. The nuclei were counterstained for visualization with 4′, 6- diamidino-2-phenylindole. The primary antibodies used were anti-Stro-1 (R&D Systems, Minneapolis, MN), anti-CD34 (Cell Signaling Technology), anti-SM22-α (Abcam, Cambridge, MA), anti-ER-TR7 (Santa Cruz Biotechnology), anti-CD68 (Abcam), anti-Notch1 (Santa Cruz Biotechnology), anti-activated Notch1 (Abcam), anti-Jagged1 (Santa Cruz Biotechnology), anti-Delta (Santa Cruz Biotechnology), and anti-Hes1 (EMD Millipore). Table 2 provides detailed information on the primary antibodies. The secondary antibodies used were Alexa Fluor 488-, Alexa Fluor 568-, and Alexa Fluor 647-conjugated anti-immunoglobulin G (Invitrogen, Carlsbad, CA). Slides treated with normal immunoglobulin G only were used as negative controls. Quantification of the staining results was performed by randomly selecting 4 fields in each slide (n = 4 in each group), and counting target cells at a magnification of ×600 using the software Image-Pro Plus 6.0 (Media Cybernetics, Bethesda, MD).

### Statistical analysis

All quantitative data are presented as the mean ± standard deviation. Data were analyzed with SPSS software, version 11.0 (SPSS Inc, Chicago, IL). The differences among the 3 groups were evaluated by Pearson's chi-squared test for categorical variables and by one-way analysis of variance for continuous variables. Two-tailed probability values are reported.

## Results

### Overall activation of Notch signaling is increased in the aortic wall of DTAAD patients

**Figure 1:**
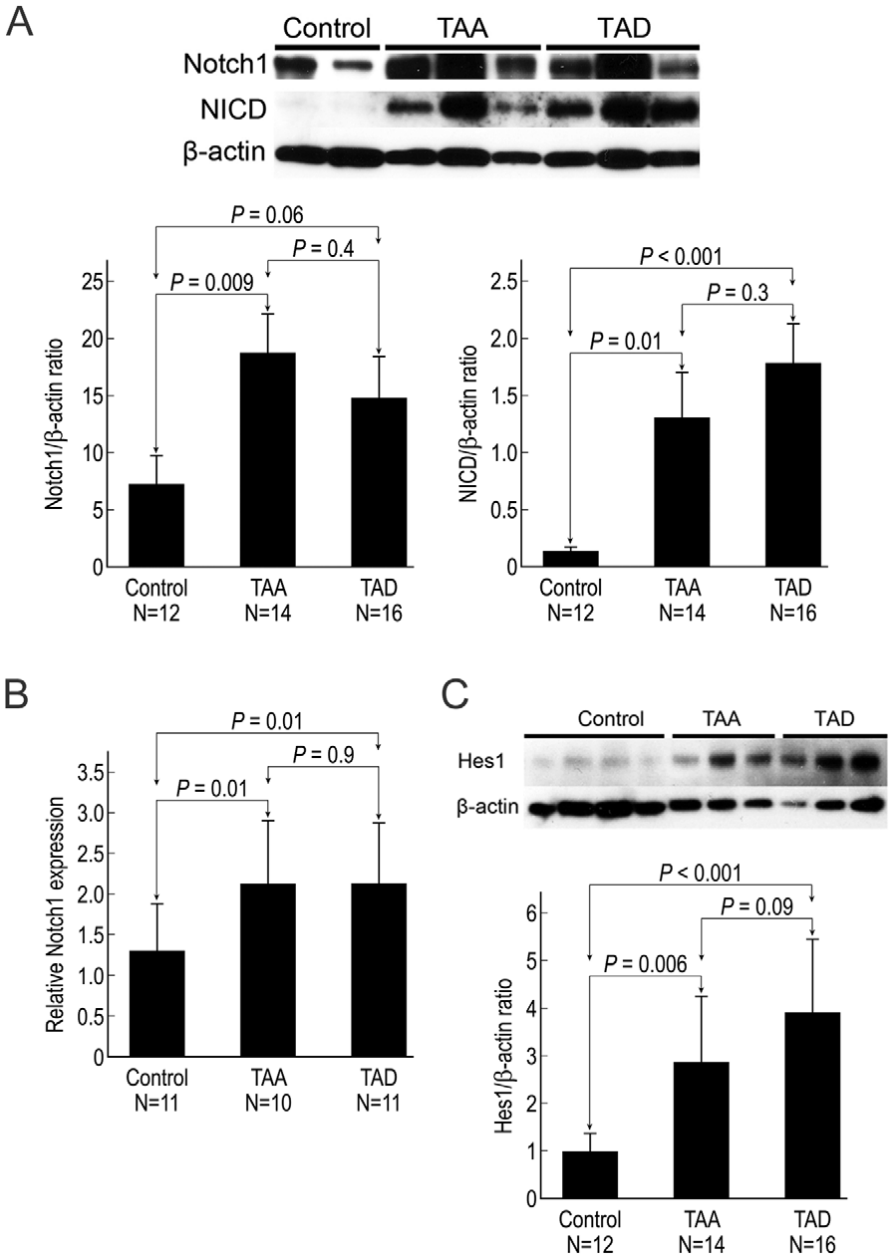
Overall activation of Notch signaling is increased in the aortic wall of DTAAD patients. A) Western blot studies showed that the expression of Notch1 (transmembrane/intracellular region NTM, ∼120 kDa) in human aortic tissue was significantly increased in TAA tissue compared with control tissue, and NICD was significantly increased in TAA and TAD samples compared with control. B) Quantitative real-time RT-PCR showed increased expression levels of Notch1 in TAA and TAD samples compared with control. C) Western blot studies showed that the expression of Hes1 in human aortic tissue was significantly increased in TAA and TAD samples compared with control.

To examine the activation of Notch signaling in the aortic wall, we performed western blots on the protein lysate from aortic tissues. The level of the Notch1 protein (transmembrane/intracellular region NTM, ∼120 kDa) was significantly increased in the aortic wall of TAA patients compared with control patients (P = 0.009);the levels were higher in TAD patients than in controls, but that difference did not reach statistical significance (P = 0.06) (Fig. 1A). Although the full-length version of the Notch1 protein (∼300 kDa) was not detected via western blot, real-time RT-PCR showed increased levels of Notch1 mRNA in TAA and TAD tissues (Fig. 1B), indicating that the upregulation of Notch1 may be at the transcriptional level. Additionally, NICD, the active form of Notch, was barely detectable in the aortic tissue of controls but was highly expressed in both TAA and TAD tissues (Fig. 1A). Furthermore, Hes1, which is a downstream target of Notch signaling, was also significantly increased in TAA and TAD samples (Fig. 1C). Together, these findings indicate activation of the Notch signaling pathway in TAA and TAD.

### Notch signaling is downregulated in aortic medial VSMCs of DTAAD patients

**Figure 2:**
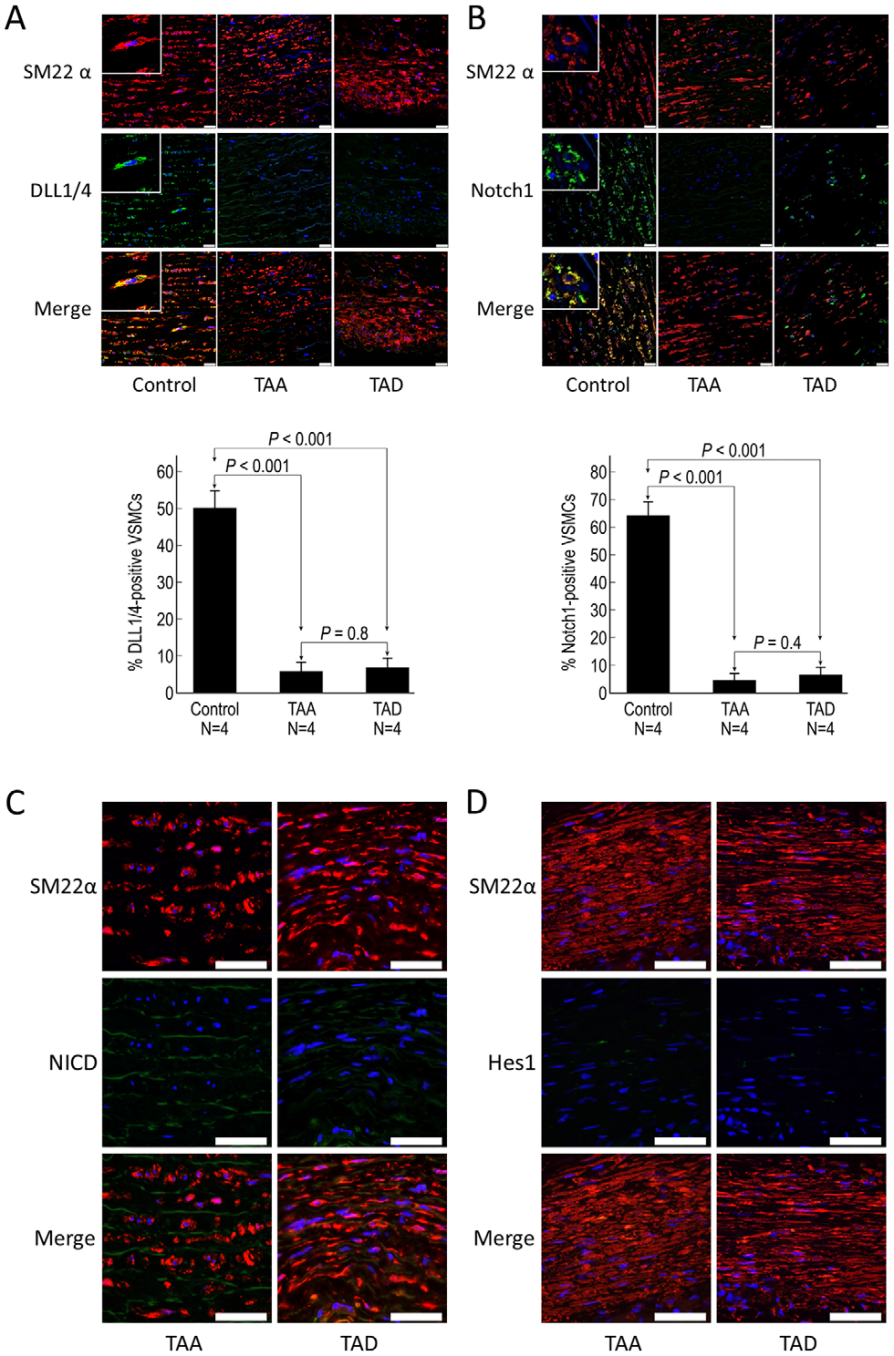
Notch signaling is downregulated in aortic medial VSMCs in DTAAD patients. A) Immunofluorescence double staining showed that DLL1/4 in VSMCs of the aortic media was significantly decreased in both TAA and TAD tissues compared with control tissue. B) Notch1 in VSMCs of the aortic media was significantly decreased in both TAA and TAD tissues compared with control tissue (scale bar = 25 µm, insets 6.25 µm). C) NICD was rarely detected in vascular smooth muscle cells (VSMCs) of the aortic media in TAA and TAD tissues. D) Hes1 was rarely detected in VSMCs of the aortic media in TAA and TAD tissues (scale bar = 50 µm).

We examined the activation of Notch signaling in different types of cells found within the aortic wall. First, we assessed Notch signaling in medial VSMCs. Immunofluorescence double staining experiments showed that both DLL1/4 Notch ligand and the Notch1 receptor were significantly decreased in the VSMCs of TAA and TAD tissues compared with control tissues (Fig. 2A, B). The active NICD and the downstream target Hes1 were rarely detected in medial VSMCs of TAA and TAD tissues (Fig. 2C, D), indicating minimal activation of Notch signaling in these cells. These findings suggest reduced production of the DLL1/4ligand and the Notch1 receptor, along with decreased Notch signaling in medial VSMCs in DTAAD tissue.

### Notch signaling is activated in CD34+ stem cells and Stro-1+ stem cells in DTAAD patients

**Figure 3:**
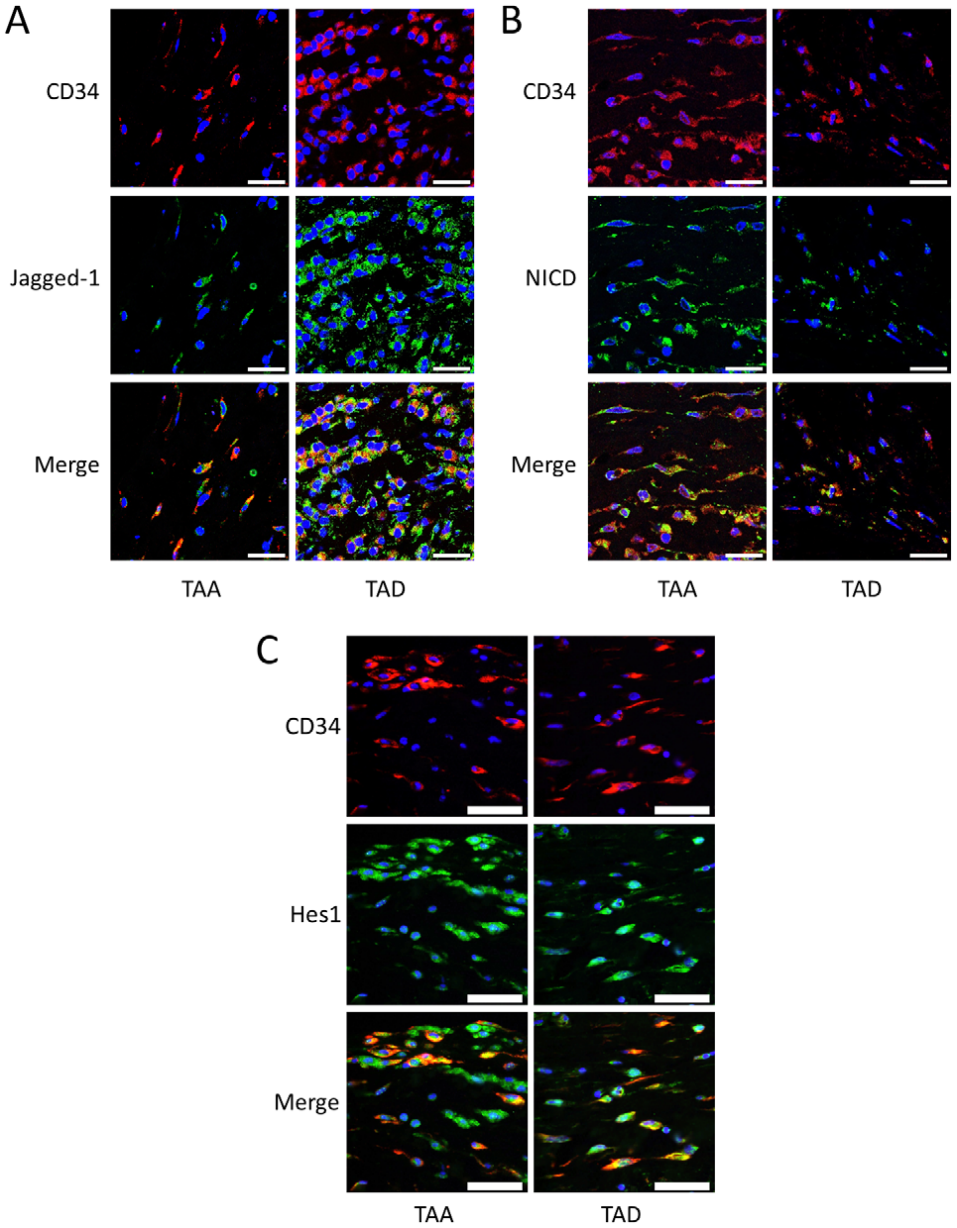
Notch signaling is activated in CD34+ stem cells in DTAAD patients. A) Immunofluorescence double staining showed that Jagged1 was expressed in CD34+ stem cells in the aortic media of both TAA and TAD tissues. B) Immunofluorescence double staining showed that NICD was highly expressed in CD34+ stem cells in the aortic media of both TAA and TAD tissues (scale bar = 25 µm). C) Immunofluorescence double staining showed that Hes1 was highly expressed in CD34+ stem cells in the aortic wall of both TAA and TAD tissues (scale bar = 50 µm).

**Figure 4:**
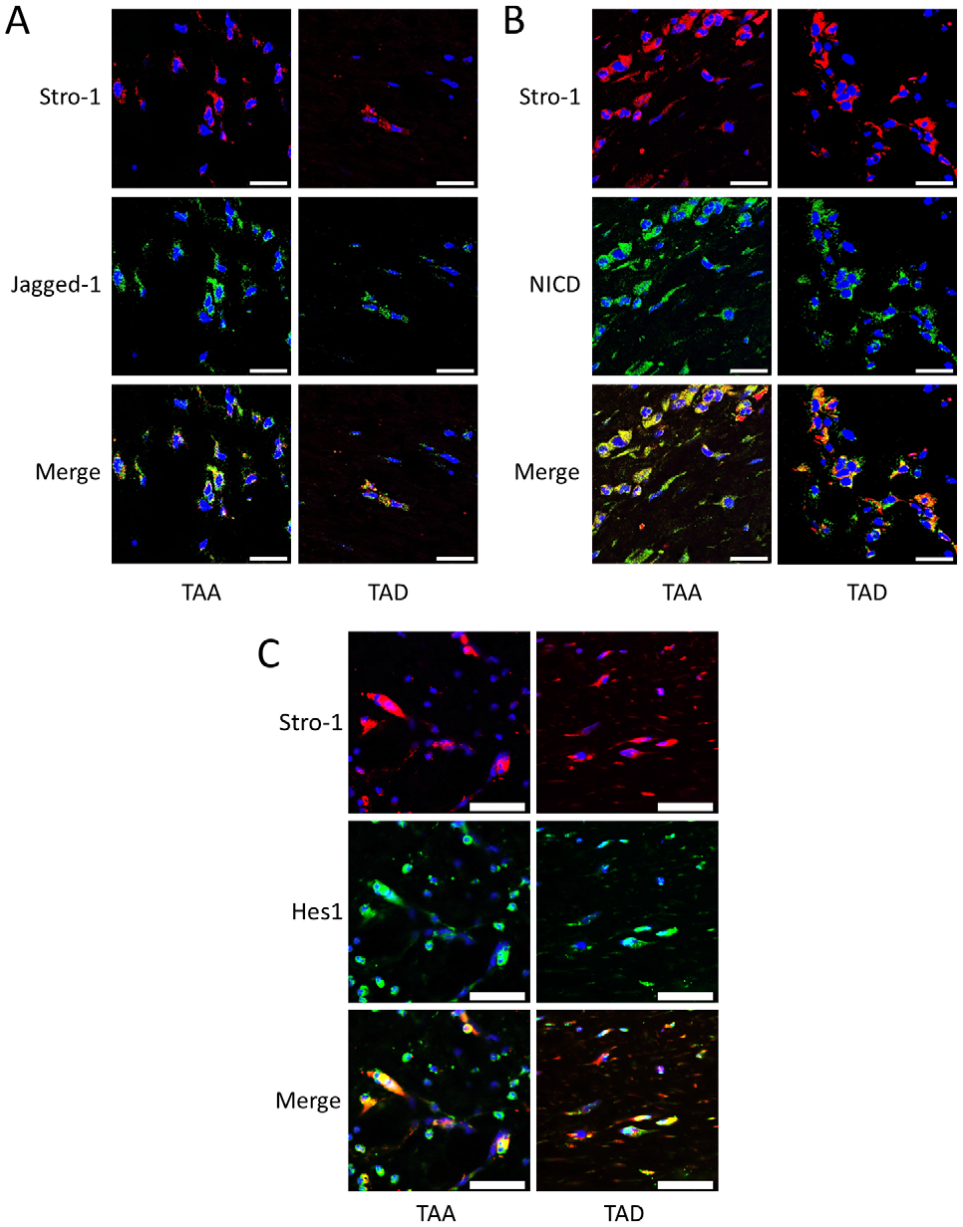
Notch signaling is activated in Stro-1+ stem cells in DTAAD patients. A) Immunofluorescence double staining showed that Jagged1 was expressed in Stro-1+ stem cells in the aortic media of both TAA and TAD tissues. B) Immunofluorescence double staining showed that NICD was highly expressed in Stro-1+ stem cells in the aortic media of both TAA and TAD tissues (scale bar = 25 µm). C) Immunofluorescence double staining showed that Hes1 was highly expressed in Stro-1+ stem cells in the aortic wall of both TAA and TAD tissues (scale bar = 50 µm).

We have previously shown that the number of stem cells was increased in TAA and TAD tissues compared to normal aortic tissue [22]. Because Notch signaling plays a critical role in stem cell proliferation [9] and SMC differentiation [13], we examined Notch activation in aortic stem cells. Double staining immunofluorescence experiments showed that Jagged1 ligand, NICD, and Hes1 were highly expressed in CD34+ stem cells (Fig. 3) and Stro-1+ stem cells (Fig. 4) in aortas from TAA and TAD patients, indicating activation of Notch signaling in these stem cells within the injured aortic wall.

### Notch signaling is activated in fibroblasts in DTAAD patients

**Figure 5:**
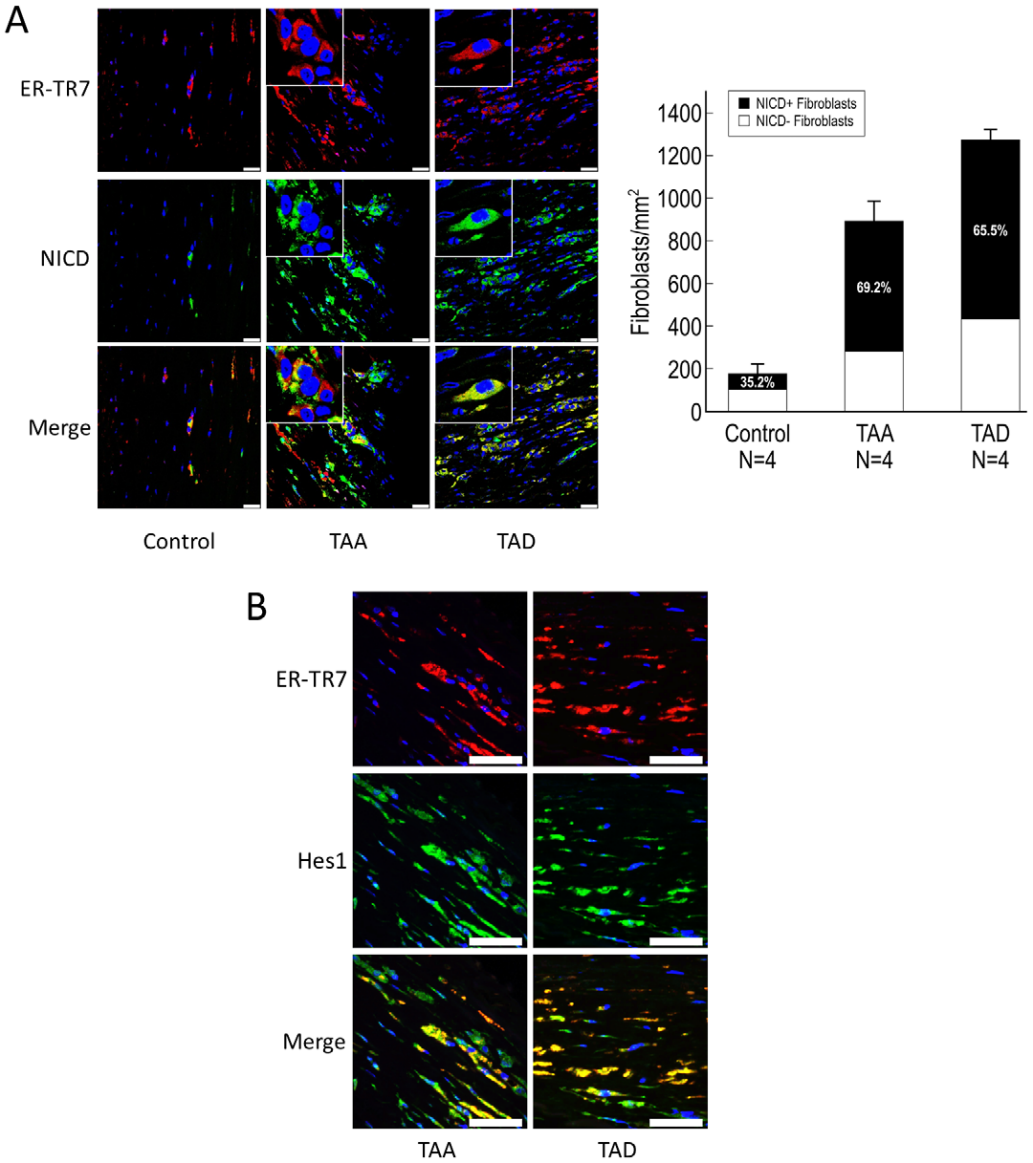
Notch signaling is activated in fibroblasts in DTAAD patients. A) ER-TR7 was used as the marker for fibroblasts in immunofluorescence double staining experiments. Significantly more fibroblasts were seen in the adventitia of the aortic wall of TAA and TAD tissues than in control tissue (TAA vs. control, P<0.001; TAD vs. control, P<0.001), and NICD was detected in most fibroblasts in TAA and TAD tissues (TAA vs. control, P = 0.009; TAD vs. control, P = 0.02) (scale bar = 25 µm, insets 6.25 µm). Error bars indicate the standard deviation in the number of NICD+ fibroblasts. B) Immunofluorescence double staining showed that Hes1 was highly expressed in fibroblasts in the aortic wall of both TAA and TAD tissues (scale bar = 50 µm).

Fibroblasts can proliferate rapidly in response to injury and contribute to tissue repair [23]. Furthermore, fibroblasts are important in maintaining aortic tensile strength and preventing aortic dilatation and rupture in response to aortic injury. Notch signaling has been shown to be involved in fibroblast-mediated tissue repair [24]. Thus, we examined changes in fibroblast levels in the diseased [24] aortic wall and the activation of Notch signaling in fibroblasts. Using ER-TR7 as a fibroblast marker, we detected significantly more fibroblasts in the adventitia of TAA and TAD tissues than in control tissue (P<0.001) (Fig. 5). Additionally, NICD was detected in most aortic fibroblasts in TAA and TAD tissues (35.2% in control; 69.2% in TAA [P = 0.009 vs. control]; 65.5% in TAD [P = 0.02 vs. control]) (Fig. 5A); Hes1 was also highly expressed in most fibroblasts (Fig. 5B). These findings indicate the activation of Notch signaling in fibroblasts of the aortic wall in DTAAD patients.

### Notch signaling is activated in macrophages in DTAAD patients

**Figure 6:**
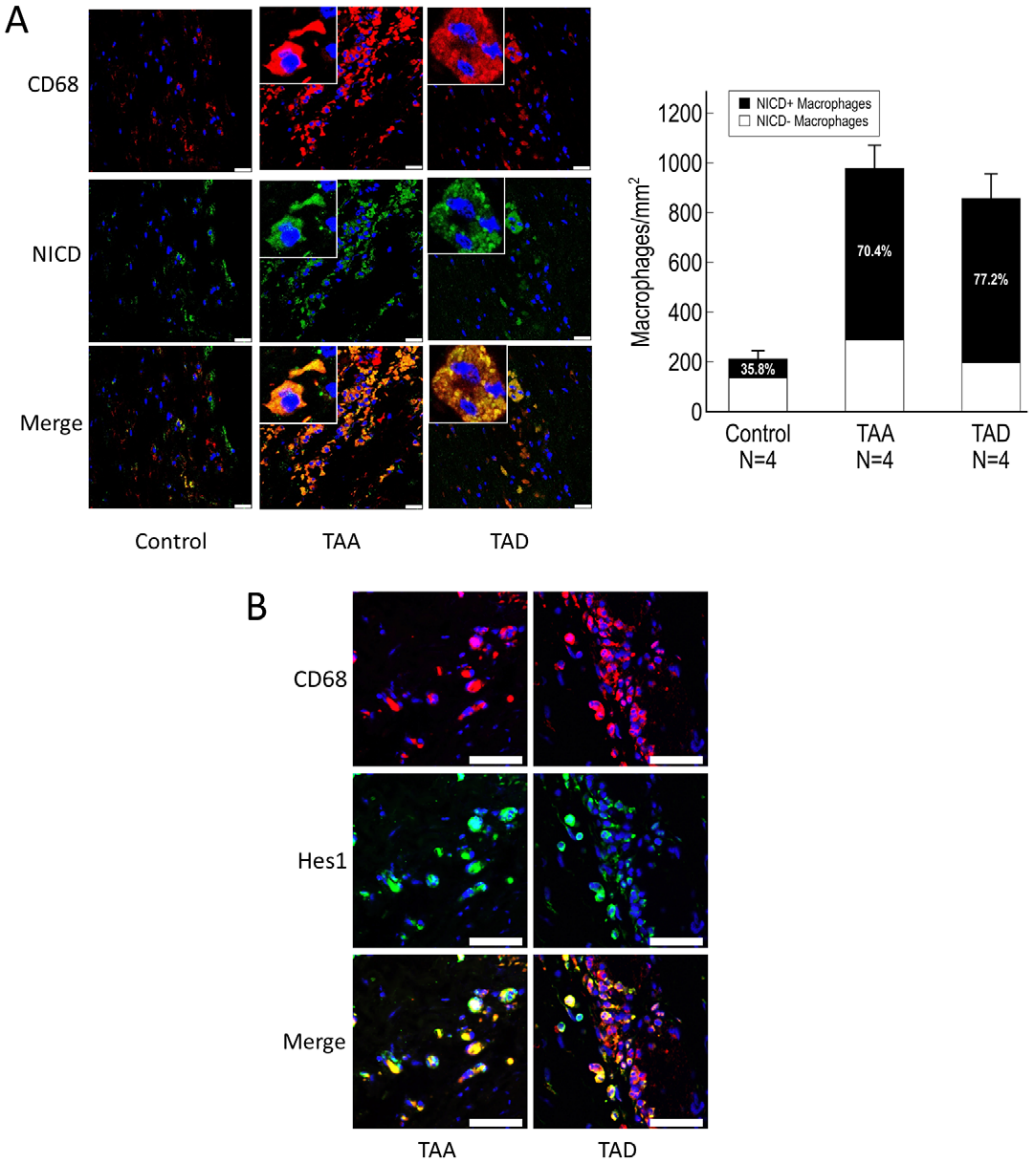
Notch signaling is activated in macrophages in DTAAD. A) CD68 was used as the marker for macrophages in immunofluorescence double staining experiments. Significantly more macrophages were seen in the aortic wall of TAA and TAD tissues compared with control tissue (TAA vs. control, P<0.001; TAD vs. control, P<0.001), and NICD was detected in most macrophages in TAA and TAD tissues (TAA vs. control, P<0.001; TAD vs. control, P<0.001) (scale bar = 25 µm, insets 6.25 µm). Error bars indicate the standard deviation in the number of NICD+macrophages. B) Immunofluorescence double staining showed that Hes1 was highly expressed in macrophages in the aortic wall of both TAA and TAD tissues (scale bar = 50 µm).

Macrophages have been shown to play a critical role in aortic destruction and AAD development [25], [26]. Moreover, Notch1 positively regulates IL-6 expression in activated macrophages [26]. Thus, we examined Notch activation in macrophages in TAA and TAD. Using CD68 as the marker for macrophages, we found significantly more macrophages in the aortic wall in TAA and TAD tissues than in control tissue (P<0.001). Moreover, NICD was detected in most macrophages in TAA and TAD tissues (35.8% in control; 70.4% in TAA [P<0.001 vs. control]; 77.2% in TAD [P<0.001 vs. control]) (Fig. 6). Furthermore, Hes1 was also expressed by most macrophages (Fig. 6B). These results suggest activation of the Notch signaling pathway in macrophages of the aortic wall in TAA and TAD patients.

## Discussion

The Notch signaling pathway is a versatile regulator of cell growth and differentiation [27], [28], [29], as well as cardiovascular development [5], [6] and repair [30]. In this study, we have shown a complex pattern of Notch signaling in the aortic tissue of patients with DTAAD. Specifically, the Notch signaling pathway was downregulated in medial VSMCs but activated in CD34+ stem cells, Stro-1+ stem cells, fibroblasts, and macrophages. Our findings suggest that impaired Notch signaling in VSMCs may contribute to the apoptosis and depletion of VSMCs that characterize DTAAD. The activation of Notch signaling in Stro-1+ stem cells, CD34+ stem cells, and fibroblasts indicates a potential role of Notch signaling in the regenerative response and remodeling process in the aortic wall. In contrast, activation of Notch signaling in macrophages suggests a role for Notch signaling in aortic inflammation during DTAAD formation and progression.

Although our study showed increased overall activation of Notch signaling in the aortic wall of DTAAD patients, we found that Notch signaling was significantly downregulated in medial VSMCs. As the main cell type in the aortic media, VSMCs are critical for maintaining aortic structure and function. Apoptosis and depletion of VSMCs are common features of AAD [31]. Deficient repair or replacement of damaged VSMCs may lead to impaired aortic healing and AAD formation. Notch signaling has been shown to promote aortic SMC proliferation and inhibit apoptosis [27]. Furthermore, it is well known that Notch signaling promotes VSMC differentiation [28], [32], [33] and regulates SMC functions [34], [35] by inducing various SMC genes. Thus, significant downregulation of Notch signaling in medial SMCs may be partially responsible for the SMC apoptosis, insufficient SMC repair, SMC depletion, and medial degeneration in AAD.

Multipotent stem cells play an important role in arterial repair and remodeling after injury. Circulating endothelial progenitor cells have been reported in a murine model of AAA [36] and in patients with AAA [37] or ascending aortic aneurysms [37], [38]. In a previous study, we showed that Stro-1+ and CD34+ stem cells were abundant in DTAAD [22]. Stem cell proliferation and differentiation into SMCs may be critical for aortic repair. Notch signaling has been shown to promote stem cell proliferation [9], [39]. Furthermore, upregulation of Jagged1 (and thus Notch activation) appears to be involved in the differentiation of stem cells along the SMC lineage [13]. In the present study, we found that Notch signaling was activated, and the Notch ligand Jagged1 was highly expressed in stem cells. Therefore, it is possible that activation of the Notch signaling pathway in Stro-1+ and CD34+ stem cells facilitates stem cell proliferation and differentiation into SMCs, contributing to aortic repair in DTAAD; further studies are needed to examine this potential mechanism.

Fibroblasts are important components of the aortic wall and may play diverse roles in aortic repair, remodeling, and inflammation, but the role of fibroblasts in the pathogenesis and development of AAD is poorly understood. In the present study, we observed large numbers of fibroblasts in the aortic wall of DTAAD patients. Because fibroblasts can proliferate rapidly in response to injury and thus help significantly in cardiovascular repair [23], [24], [40], our finding of large numbers of fibroblasts may represent a response to aortic injury; this response may be an attempt to help maintain aortic strength and prevent aortic dilatation and rupture. However, uncontrolled proliferation of fibroblasts promotes fibrotic remolding [41] with decreased contractile function and compliance. Additionally, fibroblasts produce cytokines and monocyte chemotactic protein-1 [42] and promote inflammatory cell recruitment/activation and aortic inflammation, all of which cause further tissue damage. Thus, proper control of fibroblast homeostasis in the aortic wall is critical. Notch signaling induces fibroblast proliferation [24], and in the present study, we observed high levels of NICD and Hes1 in most fibroblasts in TAA and TAD tissues, indicating the activation of Notch signaling. This activation may contribute to fibroblast proliferation. Further studies are required to define the role of fibroblasts in aortic remodeling during AAD formation and progression and to identify how Notch signaling regulates the process.

Macrophages play a destructive role in AAD formation and progression. Previous studies have shown that AAA expansion is associated with macrophage accumulation in regions of medial disruption, predominantly on the adventitial aspect [43]. Moreover, macrophage-mediated vascular inflammation can lead to aortic dissection [42] and contributes to aortic aneurysm formation [44]. In addition, macrophages are the major source of protease activity in aneurysmal tissues [25] and produce pro-inflammatory cytokines such as IL-6 [26]. In our study, we found significantly more macrophages in the aortic wall of TAA and TAD tissue than in control aortic tissue, and both NICD and Hes1 were detected in most macrophages; these findings indicate that Notch signaling is activated in macrophages. It was recently reported that inflammatory macrophage polarization was promoted by transcription factor IRF8, which is regulated by Notch signaling [45], and the activation of Notch signaling in macrophages positively regulates IL-6 expression via NF-κB [26]. Furthermore, blocking the Notch signaling pathway inhibits vascular inflammation in large-vessel vasculitis [46]. Thus, we speculate that the activation of Notch signaling in macrophages of DTAAD tissue contributes to aortic inflammation and plays a destructive role in DTAAD formation and progression.

In our study, the total levels of Notch1, NICD, and Hes1 were increased in the aortas of DTAAD patients. Although Notch signaling was downregulated in medial VSMCs, it was activated in CD34+, Stro-1+ stem cells, CD68+ macrophages, and EN-TR7+ fibroblasts. Because the number of these types of cells is significantly increased in DTAAD compared with control tissues, the significant activation of Notch signaling in these cell populations may be responsible for the overall upregulation of Notch signaling in the aorta of DTAAD patients.

Given the differences in the pathobiology of aneurysmal disease in a given segment of the aorta, it is unclear whether Notch signaling will show similar patterns of disease occurring in the ascending aorta, descending thoracic aorta, and abdominal aorta. It was recently reported that expression of *NOTCH3* mRNA was decreased in human AAA tissue when compared with control tissue samples from significantly younger organ donors (AAA patients, 71.5±7.5 years; donors, 42.7±12.5 years) [19]. Notch signaling was also found to be decreased in ascending aortas from patients with BAV, which increases the risk of premature death and of developing AAD, compared with patients who have a normal trileaflet aortic valve (TAV) [20]. Furthermore, genetic variation in *NOTCH1* appears to confer susceptibility to ascending aortic aneurysm formation in patients with BAV but not in patients with TAV [21]. Thus, altered Notch signaling may play a critical role in the development of AAD.

This study has several important limitations. First, it is a descriptive study, and the role of Notch signaling in aortic remodeling remains to be determined by additional studies. Second, the samples we examined represent end-stage disease, so we were unable to study the activation of Notch signaling during the early stages of DTAAD formation. Third, we observed significant variation in Notch levels, reflecting the heterogeneity of pathogenesis and disease progression in aortic tissue.

In conclusion, the Notch signaling pathway was downregulated in medial VSMCs but activated in CD34+ stem cells, Stro-1+ stem cells, fibroblasts, and macrophages. Further studies are required to determine the role of Notch signaling in each cell type and how the pathway is regulated. Moreover, understanding how to selectively regulate Notch signaling (ie, stimulate it in SMCs and inhibit it in inflammatory cells) may promote Notch-mediated aortic repair and reduce Notch-mediated aortic inflammation. This information may be useful in developing new treatment strategies for AAD.
